# Carotid atherosclerotic plaques standardised uptake values: software challenges and reproducibility

**DOI:** 10.1186/s13550-017-0285-0

**Published:** 2017-04-28

**Authors:** Nicola Giannotti, Martin J. O’Connell, Shane J. Foley, Peter J. Kelly, Jonathan P. McNulty

**Affiliations:** 1 Radiography and Diagnostic Imaging, School of Medicine, University College Dublin, Dublin, Ireland; 2 Department of Radiology, Mater Misericordiae University Hospital, Dublin, Ireland; 3 School of Medicine, University College Dublin, Dublin, Ireland; 4 Neurovascular Clinical Science Unit, Stroke Service and Department of Neurology, Mater University Hospital, Dublin, Ireland

**Keywords:** ^18^F-FDG, Analysis, Carotid atherosclerotic plaque, Positron emission tomography–computed tomography

## Abstract

**Background:**

Positron emission tomography–computed tomography (PET-CT) carotid standardised uptake values (SUV) of ^18^F-fluorodeoxyglucose (^18^FDG) have been proposed as an inflammatory biomarker for determining cerebrovascular diseases such as stroke. Consideration of varying methodological approaches and software packages is critical to the calculation of accurate SUVs in cross-sectional and longitudinal patient studies. The aim of this study was to investigate whether or not carotid atherosclerotic plaque SUVs are consistent and reproducible between software packages.

^18^FDG-PET SUVs of carotids were taken in 101 patients using two different software packages. Quality assurance checks were performed to standardise techniques before commencing the analysis where data from five to seven anatomical sites were measured. A total of ten regions of interest were drawn on each site analysed. Statistical analyses were then performed to compare SUV measurements from the two software packages and to explore reproducibility of measurements. Lastly, the time taken to complete each analysis was measured and compared.

**Results:**

Statistically significant differences in SUV measurements, between the two software packages, ranging from 9 to 21.8% were found depending on ROI location. In 79% (*n* = 23) of the ROI locations, the differences between the SUV measurements from each software package were found to be statistically significant. The time taken to perform the analyses and export data from the software packages also varied considerably.

**Conclusions:**

This study highlights the importance of standardising all aspects of methodological approaches to ensure accuracy and reproducibility. Physicians must be aware that when a PET-CT data set is analysed, subsequent follow-ups must be verified, if possible, with the same software package or cross-calibration between packages should be performed.

## Background

The World Health Organization (WHO) considers cardiovascular diseases as the leading cause of global death, with 17.3 million deaths reported in 2008 and stroke accounting for 6.2 million of them [1, 2]. Atherosclerosis is recognised as the major pathophysiological process which underlies most cardiovascular diseases including transient ischaemic attacks (TIA) and strokes. To date atherosclerotic plaque stratification and treatment are based on the portion of the lumen occluded, however, this is now being questioned by several studies that linked the likelihood of developing cerebrovascular events with the inflammation status and vulnerability of each plaque rather than to their morphology [3–6].

^18^F-fluorodeoxyglucose (^18^FDG) positron emission tomography–computed tomography (PET-CT) has recently attracted considerable interest due to its ability to depict the inflamed and vulnerable status of atherosclerotic plaques [7, 8]. ^18^FDG is a glucose analogue which is taken up by the inflamed plaque, due to their high macrophage activity and associated gene expression, and once the radiolabelled tracer is injected inside the body, the C-2 hydroxyl group in the glucose molecule is substituted by the positron-emitting radionuclide ^18^F [9–12].

Unlike in oncology, where PET is widely accepted and well validated [13], the use of ^18^FDG PET-CT imaging of atherosclerotic plaque represents a relatively novel area in nuclear medicine since its feasibility and clinical use are still under investigation. Despite the fact that hypermetabolic areas and higher SUVs measured on PET images, and verified with histological findings, have been associated with macrophage activity and plaque development, no fixed criteria have yet been proposed to associate an increase in SUV with the likelihood of developing vulnerable plaque. The latter consideration suggests that standardised SUV measurements may play a critical role when the metabolic activity of plaque is evaluated. As previously explored by Adams et al., SUV measurements may be influenced by technological and biological factors and, as demonstrated, it is important to perform and follow up SUV measurements using the same type of scanner [14]. However, there has been little focus on vascular PET-CT imaging.

Although differences between software packages used for oncological PET-CT measurements have been previously explored [15, 16], we hypothesised that exploring differences between SUVs of atherosclerotic plaques may present further challenges due to their different glucose metabolism, uptake time, spatial resolution and size of lesions. Given the importance being placed on plaque SUV measurements, the aim of this study is to verify whether carotid SUVs measured with different software packages that we have available in our institution are consistent and reproducible over time and between software packages.

## Methods

This study forms part of a larger, multicentre prospective cohort study exploring the relationship of carotid plaques ^18^FDG PET uptake (mean and maximum SUV) with early recurrent stroke in patients with recently symptomatic carotid stenosis. All procedures performed in this study involving human participants were in accordance with the ethical standards of the institutional committee and with the 1964 Helsinki declaration and its later amendments.

As part of this cohort study, 102 patients aged over 50 years who presented with recent (within 30 days) onset of speech or motor TIA or mild-to-moderate ischaemic stroke and non-occlusive ipsilateral carotid atherosclerosis, causing at least 50% lumen narrowing, on duplex ultrasound, CT or magnetic resonance (MR) angiography, or digital subtraction angiography were prospectively enrolled in the study between 2011 and 2016 (Table 1). Exclusion criteria for enrolment were pregnancy, significant renal impairment (estimated glomerular filtration rate <60 ml/min) or other contraindication to contrast-enhanced CT, active malignancy, dementia, unstable medical condition, prior neck irradiation, history of ipsilateral carotid surgery or endovascular stenting.

**Table 1:** Clinical characteristics of patients

Characteristic	Patients
Total number	102
Average age, years (range)	65 (58–95)
Male/female	56/46
Statin	42
Antiplatalet therapy pre-PET	41
Diabetes mellitus	13
Smoker	50
Carotid stenosis >70%	30

Although aspirin 75 to 325 mg daily was suggested for patients in BIOVASC, treatment and drug choices were at the discretion of each treating physician. In 42 cases, atorvastatin 10 mg was prescribed.

**Fig. 1:**
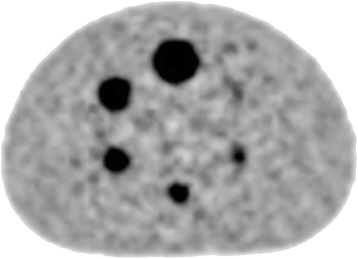
Image taken from a central slice of the NEMA Phantom

Prior to clinical PET-CT scanning patients, an image quality and SUV accuracy assessment was carried out using the NEMA NU2-2001/2007 IEC Body Phantom (Fig. 1).

The phantom consists of a body phantom and six fillable spheres filled relatively with 5 kBq/ml ^18^FDG and 25 kBq/ml ^18^FDG solutions to simulate small regions of tracer uptake (Fig. 1). Data was acquired using the same acquisition and processing parameters used for patient studies.

A full quantitative analysis was carried out, and a recovery curve was generated by comparing the actual concentration of ^18^FDG in each of the spheres with the measured concentration. The recovery curve was compared with the European Association of Nuclear Medicine (EANM) recovery curve specifications.

Following full institutional ethical approval and informed consent, all subjects underwent an ^18^FDG PET-CT scan. ^18^FDG PET-CT was performed using a Siemens Biograph 16 scanner (Siemens, Erlangen, Germany) after a minimum 6-h fast. Blood glucose level was verified for each patient and if above 11 mmol/l the PET-CT scan was not performed.

Three hundred twenty megabecquerel of ^18^FDG was administered 2 h prior to image acquisition. The uptake phase was standardized with the patient resting. PET images were acquired in 3D mode in two bed positions for 10 min each. Slice thickness of 3 mm and a 256 × 256 matrix were used. PET emission mode images were acquired and reconstructed by applying OSEM2D4i24s algorithm and XZY Gauss 2.0 convolution kernel.

A low-dose CT scan for attenuation correction was performed using the same scanner directly after PET; in addition where the administration of a contrast agent (Omnipaque 350, GE Healthcare, Milwaukee, USA) was not contraindicated (serum creatinine level >1.5 mg/dl or estimated glomerular filtration rate <60ml/min), a diagnostic carotid angiogram (CTA) was performed using bolus tracking. The pre-monitoring slice was set at the aortic arch, and a circular region of interest (ROI) was drawn distant from any vessel calcification.

CT images (3 mm slice thickness, with contrast enhancement) were acquired from the aortic arch to the skull base to identify carotid arteries and jugular veins. CT parameters were 120 kVp and 104 mAs were selected for the head and neck protocol used along with a 512 × 512 matrix, pitch of 0.6 with 1 mm CT slice reconstructions following acquisition.

Of these 102 PET-CT data collected, one case was excluded due to the presence of metallic dental implants which impacted on the image quality.

Before commencing the analysis, a series of quality assurance (QA) checks were performed for all cases using both software packages OsiriX MD® version 6.5.2 (Pixmeo © SARL, Geneve) and AquariusNet iNtuition™ version 4.4.11 (TeraRecon, Foster City, CA, USA). These checks included both co-registration and qualitative visual checks. Both software packages assessed used a semi-automatic linear (or rigid) registration algorithm to fuse PET and CT data together; therefore, computed alignment transformation between image modalities included, and were limited to, rotation, translation and scaling. Further manual spatial adjustments based on anatomical landmarks were made by a single reader in order to correctly register the two imaging modalities.

Visual checks were performed on each CT dataset to confirm the presence of stenosis within the internal carotid arteries. Maximum intensity projection (MIP) images were created, cropped and windowed to verify stenosis identified, in the first place, on axial slices.

For all data sets included in the study, the presence or absence of brown fat was considered as it would impact on SUV measurements. Hounsfield values on the CT images were assessed as brown fat would be within the range of −50 and −150 [17]. Checks were also made for hypermetabolic lymph nodes, due to active inflammation outside the structures of interest; contrast-enhanced CT images helped to identify the anatomical vascular structure avoiding adjacent hypermetabolic areas [17].

**Fig. 2:**
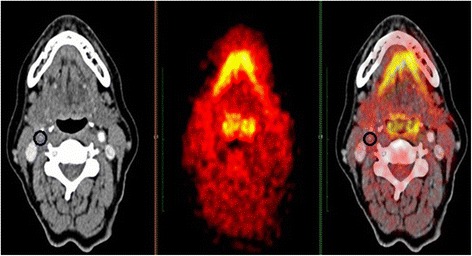
Circular region of interest (ROI) drawn on a stenosis of the right internal carotid artery

**Fig. 3:**
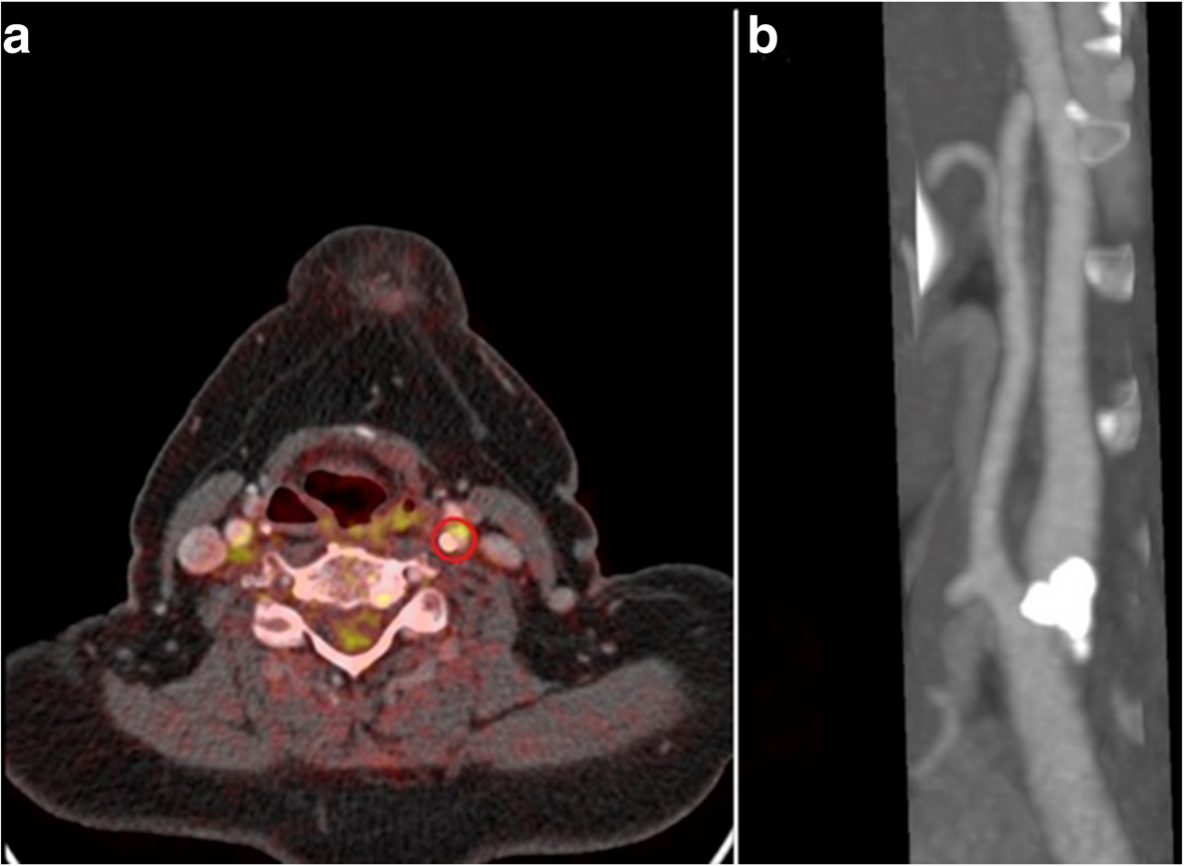
**a** The point of maximum stenosis was used as midpoint of the segment analysed (*red circle*). **b** The stenosis identified on MIP images within the *left* internal carotid artery

Once all the QA checks were performed, ten ROIs were placed on different vascular anatomical sites at 1 mm intervals:

- Common carotid artery (CCA) left and right, five circular ROIs were drawn above and below the anterior inferior margin of the larynx;
- Jugular vein, ten ROIs were drawn as per CCA right and left;
- Carotid bifurcation left and right, ten ROIs were drawn downwards from the point of bifurcation (carotid bulb);
- Internal carotid artery (ICA) left/right (Fig. 2), only if a stenosis was identified, in the ICAs, ten ROIs were drawn including the point of maximum stenosis as mid-point of the segment analysed (Fig. 3). For ICAs stenosis identification, the reader used the maximum intensity projection (MIP) algorithm through which confirming the presence of stenosis was possible (Fig. 3).

SUV body weight was selected for the analysis. Since the SUVs for vascular plaques may only differ marginally from normal uptake [4], a background ratio (the flowing blood within the jugular vein was used for this analysis) to use as an internal control was obtained.

SUV was not corrected for plasma glucose level (SUV × plasma glucose level/5.0; where 5.0 represent the population average of plasma glucose concentration); however, patients with a glucose level above 11 mmol/l were not scanned.

For each anatomical site the mean, maximum, minimum and standard deviations of the SUVs were measured by drawing circular ROIs. The axial ROIs were positioned carefully to include the full vessel wall and lumen (Fig. 2). Therefore, due to the vessels morphology, the size of the ROI drawn on each slice analysed varied between slices.

The ROI placement approach differed between the two software packages; every ROI had to be manually drawn on TeraRecon iNtuition™ while a semi-automated approach was used for ROI placement on OsiriX MD® as it automatically computes and contours the profile of a series of ROIs placed between two distal ROIs. Care was taken in the positioning of all ROIs with some edits made to ensure the inclusion of the entire vessel wall and in accordance with the anatomical landmarks described above. A consistent size of ROI was not used as vessels may change their dimension along their length. Therefore, 28 data points were collected using each software package for each patient and exported into SPSS version 21 (IBM, NY, USA) for further statistical analyses.

Data were not found to be normally distributed thus non-parametric tests including the Wilcoxon signed-rank test; the Mann–Whitney *U* test and Spearman’s rank order correlation were used. Intra-reader reliability was verified for both software packages by repeating the analysis on 20 cases per software package following a 4-week interval with the reader blinded to previous results. The intra-class correlation (ICC) test was used to assess the reliability. The time taken to complete each case’s analysis with both software packages was measured by averaging the time spent to complete the analysis on 10 cases with each software package. These time measurements considered the time spent for the image fusion process, QA checks, mis-registration issues, ROIs drawing and lastly, the measurements export phase.

## Results

Between OsiriX® and TeraRecon iNtuition™, 48,760 data points were collected and analysed (24,380 for each software package). An overall statistically significant difference in SUV measurements between software packages was found in 79% of cases when each pair of variables was compared (*p* < 0.05).

As expected, SUV maximum measurements at different vascular sites (left carotid bifurcation, right carotid bifurcation, common carotid artery left, common carotid artery right, right and left internal carotid artery), when compared with the two software packages did not demonstrate any statistically significant differences.

Differences as large as 21.8% in SUV measurements were found when the SUV minimums from the two software packages were compared, while differences were within a 9% interval when the SUV means were compared. In this last case, the maximum difference found between the two software packages was of 9% between ICAs left (1.57 for TeraRecon iNtuition™ versus 1.71 for OsiriX®).

To test whether or not a software package consistently measured higher SUVs the Mann–Whitney *U* test was performed for each couple of variables. SUV measurements were converted into mean ranks and then compared. This identified that in 79% of cases, Osirix® was found to produce higher measurements than TeraRecon iNtuition™.

The Spearman’s rank order test showed a good correlation when SUVs mean and maximum for each variable were correlated, *r*_s_ values respectively of 0.73 and 0.7 (*p* < 0.001).

The intra-reader reliability, measured with the ICC test, showed very good agreement between repeated measurements of the same sample of population using the same software after a certain interval of time (ICC, *α* = 0.864; *p* < 0.001).

In terms of time spent for analysing each case (QA, ROI drawing and data export), a considerable statistical difference was identified (*p* < 0.05); the average time spent was 25 min using OsiriX® and 1 h with TeraRecon iNtuition™.

## Discussion

Since 2002, a considerable number of papers looking at PET imaging atherosclerotic plaque with ^18^F–FDG began to be published and to be taken into consideration [5, 18]. An increasing number of research groups focused their attention on PET imaging of atherosclerotic plaque to determine the likelihood that they may have to develop cerebrovascular events.

Despite the rising need to validate new diagnostic biomarkers that may help to diagnose vulnerable atherosclerotic plaque, so far, due to the variability of PET imaging protocols used worldwide, measurements obtained through different studies could not have been compared [19].

The analysis performed and described in this article is part of a large multicentre study called BIOVASC, a cohort study in which patients with recent symptoms of cerebral ischaemia and stroke causing no greater than moderate disability and evidence of ipsilateral carotid stenosis causing at least a 50% narrowing are recruited and followed up for 1 year (Fig. 3). One aim of BIOVASC is to independently validate the relationship of carotid plaque ^18^FDG PET uptake with early recurrent stroke. In our earlier work, ^18^FDG PET-CT imaging of atherosclerotic plaque was found to be a valid prognostic tool to predict patients to whom stroke or ischaemic attacks are more likely to happen within 90 days from the first onset event [3].

The aim of this analysis was to provide an insight into carotid atherosclerotic plaque PET-CT analysis using different software packages that are available in our institution. Furthermore, several QA checks were explored to define a standardised reliable and reproducible technical approach for carotid plaque analysis. To date, several studies have been published looking at variability in SUV measurements; however, to date, no studies have looked into differences between PET-CT software packages when carotid atherosclerotic plaque data obtained through the same scanner are analysed.

In PET, inter-scanner variability is a critical consideration in cross-sectional and longitudinal studies. This also holds through for the approaches used to establish SUVs. Our results demonstrate statistically significant differences between all variables analysed with the exception of SUV maximum measurements. SUV maximum measurements represent the value of the highest voxel within the ROI and therefore are not volume/area dependent.

SUV maximum is currently the most common SUV indicator used as it has been found to be more reproducible and less operator dependant; however, it is more susceptible to noise [14].

Although a significant difference was found (within a 9% interval) when SUV means were compared, results showed a good statistical correlation between measurements. The main reason behind variability in SUV means may be associated with the difference in ROI size as the SUV mean includes information regarding the average uptake value of all pixels within a ROI. Therefore, its value varies depending on which pixels are included, thus making it susceptible to inter and intra-reader variability.

In this study two-dimensional (2D) circular ROIs were placed on five to seven standardised anatomical sites; each ROI was manually positioned when data were analysed with TeraRecon iNtuition™, while they were positioned using a semi-automatic approach when date were analysed with Osirix MD®. For this reason, variability in ROI sizes might have been introduced between the two software analyses.

Although not used in clinical practice, SUV minimum measurements were obtained as part of the protocol showing the largest difference when data from Osirix MD® and TeraRecon iNtuition™ were compared; significant differences as large as 21% were found in SUV minimum measurements. Despite the fact that the reproducibility of SUVs is affected by ROI size, SUV measurements obtained from large and fixed ROIs have been shown to be highly reproducible [20, 21]. The ICC test performed on SUV measurements of small atherosclerosis lesions demonstrated a good degree of reproducibility due to the carefully designed methodological analysis (*α* = 0.864; *p* < 0.001).

For this reason, any cross-sectional or longitudinal study that involves more than one centre, a detailed methodological analysis along with appropriate QA steps must be agreed and in place in order to standardise protocols, reduce operator variability and other undesirable drawbacks.

Differences in SUV measurements between software packages may be due to different post-processing algorithms applied by different vendors to their images before SUV computation [15]. Or indeed, different vendors may apply different filters as described by Kelly and Declerck [22] in relation to an anisotropic three-dimensional (3D) Gaussian filter applied as part of the pre-processing phase.

Furthermore, an additional source of variability introduced in our analysis which is important to discuss relates to the registration technique that software packages apply when different image data sets are fused. Image registration is an important tool that facilitates the direct comparison between anatomical information and glucose metabolism in ^18^FDG PET-CT. Two different registration techniques are currently available: linear or rigid and deformable. Both TeraRecon iNtuition™ and OsiriX MD® used the linear (or rigid) registration technique. The latter allows fusion of different imaging modality data sets; however, the computed alignment is limited to translation, rotation and scaling, with no compensation for motion or patient positioning is possible. In our analysis, when PET-CT images were fused, the reader had to verify the automatic fusion process that each software package applies but for most of the cases manual spatial adjustments had to be made. These spatial adjustments were based on anatomical landmarks and thus based on the ability of the reader to correctly match the two imaging modalities. For SUV mean and maximum measurements, this subjectivity in image registration may introduce variability when data are collected. Lastly, as suggested by Slomka deformable registration techniques, preferably based on volume algorithm, should be applied when scans may require nonlinear transformation to compensate for changes in body configuration, breathing patterns or movements of internal organs [23].

The background ^18^FDG PET uptake was measured by drawing circular ROIs in the jugular vein at the same level of ROIs in the CCA right and left. This can be used to calculate the lesion-to-background ratio, and it may moreover reduce the statistical differences measured between SUVs mean and max. ^18^FDG PET background values will be used for further analysis when the patients’ outcome will be correlated with ^18^FDG PET SUVs; however, with this analysis, we only compared the SUVs obtained with two different software packages.

Statins have been seen as an important medical treatment to reduce low-density lipoprotein cholesterol (LDL-C) and increase high-density lipoprotein cholesterol (HDL-C). In 2006, Tahara et al. randomised 43 patients in two groups (statin versus dietary management) to investigate whereas statin may attenuate plaque inflammation by using ^18^FDG PET. Results showed that statin, but not diet alone, significantly reduces (*p* < 0.01) ^18^FDG plaque uptake and their relative SUVs by decreasing the total level of LDL-C and increasing the HDL-C [24].

Although statin may represent a limitation, if not an exclusion criteria in most studies conducted using ^18^FDG PET, this was not the case as our study compared SUVs obtained at the same time through two different software. Therefore, ^18^FDG PET characteristics did not change over time for the 42 patients here who used statins.

With current PET scanners, the limit of resolution for detecting lesions by ^18^FDG PET ranges between 0.4 and 1.0 cm in diameter; therefore, small lesions are difficult to identify with PET [13]. In oncology, this issue has been translated into the capability of PET scanners to identify only solid tumours bigger than 1 g (10^8^ to 10^9^ cells) [13]; however, no validation studies have been published so far looking at the limiting spatial resolution of PET for atherosclerotic lesions. Due to the generally smaller dimensions of plaque within carotid arteries, the range of 0.4 to 1.0 cm of spatial resolution must be considered as an important limitation since the SUV measured on a specific point of the plaque may have been produced from a point outside the plaque.

A major strength of combined PET-CT scanners is to acquire accurate and co-registered functional and morphologic images. With the use of the CT component to provide attenuation correction of PET dataset, the total exam time has been considerably reduced along with the noise caused by the transmission attenuation correction [25]. However, when the attenuation correction is provided by CT, Hounsfield units (HU) originating from photons with a mean energy of 100 to 140 keV must be converted into the higher PET keV values (511 keV). To date, several techniques and algorithms exist that permit the HU correction. However, when structures or drugs with higher density are encountered, these tend to overcorrect measurements. In the current study, calcified components may be present in the plaques and these are a major cause of vessel occlusion. Due to its high density, calcium may alter the HU correction process creating artefacts when attenuation corrected PET-CT images are fused and SUVs measured. Therefore, to overcome this issue, non-attenuation corrected images must be verified to identify whether or not a hypermetabolic area is seen on the same slice/area. However, since the latter issue has only been associated with calcified lymph nodes and not plaque, we excluded a priori source of artefacts from the analysis. Nevertheless, further work should be done to analyse whether or not small calcified area within carotid arteries may generate the same artefact.

For the same reason described above, even the use of iodinated contrast agents in PET-CT is controversial as it may become a source of artefact. When the contrast agent is injected into the body, an overcorrection of PET data tends to occur and could potentially mimic ^18^FDG accumulation. However, these artefacts are rare and tend to happen only in proximity of major hyper-metabolic lesions in oncology.

Having the CT data set available for further analysis (1 mm CT reconstruction), our next aim is to evaluate and potentially correlate the patients’ clinical outcome with SUVs and plaque composition assessed by CT.

In this study, an important limitation is represented by the fact that only one reader was used to perform the analysis and that despite the fact that several software are available to perform PET-CT analyses only two of them were chosen and compared. Therefore, no information regarding the inter-reader reliability are available; and moreover, we may only hypothesise that other software packages, which we did not explore but that are commercially available, may produce further differences between measurements.

This study represents a first insight and analysis into PET-CT software differences when atherosclerotic plaques are analysed, therefore for this method to become routine in cross-sectional or longitudinal studies and in clinical practice attention must be paid to SUV measurements. Our findings may suggest that the use of more than one software, for such studies or when data of the same patient are analysed, should be avoided or, if this is not possible, a margin of error which is intrinsic to the software and technique should be taken into consideration when reports are made; moreover the history of previous exams must be checked.

Another major difference between the two software packages was the time taken to complete the analysis. OsiriX® was found to be more user friendly for ROI placement and data export; given an initial and an end ROI, the software can automatically contour the missing ROIs in between the two points. Regarding the data export process, OsiriX® offers a plugin that allowed data to exported to Excel in one click, whereas the export process with TeraRecon iNtuition™ was less streamlined.

## Conclusions

This analysis provides an important step in the exploration of PET-CT as an inflammatory biomarker by providing an understanding into the performance of different software. Although differences regarding techniques and measurements exist among software packages when PET-CT data sets are analysed, this study highlights the importance of standardising all aspects of methodological approaches to ensure accuracy and reproducibility. While this study demonstrates a methodology for establishing carotid plaque SUV measurements, the statistically significant differences identified highlight the need for caution when interpreting results provided by third party software packages.
